# 

**Published:** 1846-05

**Authors:** 


*Premature Interments.*—It is stated that the cases of premature interment in France, prevented by fortuitous circumstances, amount, since the year 1833, to 94. Of these, 35 persons awoke of themselves from their lethargy at the moment the funeral ceremony was about to commence; 13 recovered in conseqnence of the affectionate care of their families; 7 in consequence of the fall of the coffins in which they were inclosed; 9 owed their recovery to wounds inflicted by the needle in sewing their winding sheet; 5 to the sensation of suffocation they experienced in their coffin; 19 to their interment having been delayed by fortuitous circumstances; and 6 to their interment having been delayed in consequence of doubts having been entertained of their death.—*Prov Med. and Surg. Journ.*

